# The use of Zuckerkandl's tubercle as an anatomical landmark in identifying recurrent laryngeal nerve and superior parathyroid gland during total thyroidectomy: a prospective single-surgeon study

**DOI:** 10.3389/fsurg.2023.1289941

**Published:** 2023-10-30

**Authors:** Ercument Gurluler

**Affiliations:** Department of General Surgery, Uludag University Faculty of Medicine, Bursa, Türkiye

**Keywords:** thyroidectomy, Zuckerkandl's tubercle, anatomical landmark, recurrent laryngeal nerve, superior parathyroid gland

## Abstract

**Objective:**

To determine the incidence and characteristics of Zuckerkandl's tubercle (ZT), and its relationship with recurrent laryngeal nerve (RLN) and the superior parathyroid gland (SPG) in the setting of total thyroidectomy.

**Methods:**

A total of 421 patients (mean (min-max) age: 45.6 (18–78) years, 76.2% were females) who had total thyroidectomy were included in this prospective single-surgeon thyroidectomy series study. Patient demographics and thyroidectomy indications (benign and malignant) were recorded in each patient. The presence, grade and laterality of ZT, and its relationship with RLN and SPG were recorded during surgery.

**Results:**

Most of the thyroidectomy indications (69.1%) were related to a malignant disease. The ZT was unrecognizable in 41(9.7%) of 421 patients. In 380 patients with identifiable ZT, the grade 2 (46.3%) ZT was the most common finding. Majority of ZTs (92.9%) were unilaterally located (right-sided: 64.9%; left-sided: 35.1%). In majority of the cases (83.2%), the RLN was found to lie medial to ZT. Overall, SPG was identified in close proximity to ZT in 66.6% of patients (Class 2 [0.5–1 cm from ZT] in 46.6% and Class 3 [<0.5 cm from ZT] in 20.0%). SPG was more likely to be identified in close proximity to ZT when the grade of ZT was higher, which was found to be located 0.5–1 cm from the ZT in 56.9% and 42.7% of grade 2 and grade 3 ZTs, respectively, and <0.5 cm from the ZT in 46.1% of grade 3 ZTs.

**Conclusion:**

In conclusion, this prospective single-surgeon thyroidectomy series study indicates the likelihood of localizing the RLN medial to ZT, and the SPG in close proximity to ZT during total thyroidectomy operations. Hence, the ZT can be used as a reliable and constant landmark to localize both the RLN and the SPG during thyroid surgery, which enables minimizing the risk of iatrogenic injury to RLN, while ensuring a parathyroid-sparing thyroidectomy. The thyroid surgeon should have complete knowledge of thyroid gland anatomy and embryogenesis and should follow a careful and meticulous approach particularly for dissections around larger ZTs, given the increased likelihood of SPG and RLN to be in close proximity.

## 1. Introduction

Thyroid surgery mandates the surgeons’ expertise and in-depth knowledge of the surgical anatomy and embryogenesis, due to considerable risk of complications such as injury to the recurrent laryngeal nerve (RLN), superior laryngeal nerve, or the parathyroid glands which may result in profound lifelong consequences for the patients (1–4).

The RLN shows great variability in its thickness, route and distal ramification (5, 6). In addition, rarely it may also be atypical in the form of the non-recurrent laryngeal nerve (NRLN), which occurs at an incidence ranging from 0.3% to 1.6% for the right and approximately 0.04% for the left, as considered to be by far more prone to intraoperative injury than the RLN (5–8). Hence, proper identification and preservation of the RLN and consideration of the potential variations are important for the safety of surgical procedures of the neck (9).

The direct inspection and complete exposure of RLN during thyroidectomy using the crucial anatomical landmarks such as inferior thyroidal artery, the ligament of Berry, thyroid cartilage and the Zuckerkandl's tubercle (ZT), has become the gold standard method to prevent the inadvertent injury to the nerve, while the parathyroid-sparing surgery is a routinely applied procedure in thyroidectomies to prevent the postoperative hypoparathyroidism (3, 4, 10–14).

The ZT, named by the Austrian anatomist Emil Zuckerkandl in 1902, is a posterolateral projection of the thyroid lobes, indicating the point of fusion between the ultimobranchial body and the main median thyroid process formed during the embryogenesis (15–17). ZT presents as a lateral projection from the lateral thyroid lobe with a close relationship to the extra-laryngeal termination of the RLN as well as to the superior parathyroid gland (SPG) (17–21).

Hence, serving a useful anatomical landmark for the location of both the RLN and the SPG, ZT is often described as “friend” of a thyroid surgeon, while its recognition is also considered important to ensure the complete removal of all thyroid tissue during total thyroidectomy (14, 17, 18, 20–26). Nonetheless, there is vast heterogeneity in the incidence, size and laterality of ZT reported in literature and its utility as a landmark in thyroid surgery has not been extensively investigated, particularly in terms of identifying SPG (1, 3, 4, 14, 17, 22, 23, 26).

This prospective single-surgeon study aimed to determine the incidence and characteristics of ZT and its relationship with RLN and SPG in the setting of total thyroidectomy, and thereby to evaluate its utility as a landmark to safely locate both the RLN and SPG during thyroid surgery.

## 2. Materials and methods

### 2.1. Study population

A total of 421 patients (mean (min–max) age: 45.6 (18–78) years, 76.2% were females) who had total thyroidectomy accompanied with intraoperative exposure of RLN and SPG in relation to ZT were included in this prospective single-surgeon thyroidectomy series study conducted at a tertiary care general surgery clinic between June 2023 and June 2024. Patients who underwent thyroidectomy operations other than total thyroidectomy and those without intraoperative identification of RLN and/or SPG were excluded from the study.

The study was conducted in accordance with the ethical principles stated in the “Declaration of Helsinki” and approved by the Uludag University Clinical Research Ethics Committee (Date: 27/09/2019, Protocol No: 2019-15/1).

### 2.2. Assessments

Patient demographics (age, gender) and thyroidectomy indications (benign and malignant) were recorded in each patient. All thyroidectomy operations were carried out by the same surgeon (E.G.). The presence, grade and laterality of ZT and its relationship with RLN and SPG were recorded during thyroid surgery.

### 2.3. Presence, grading and laterality of ZT

**Figure 1:**
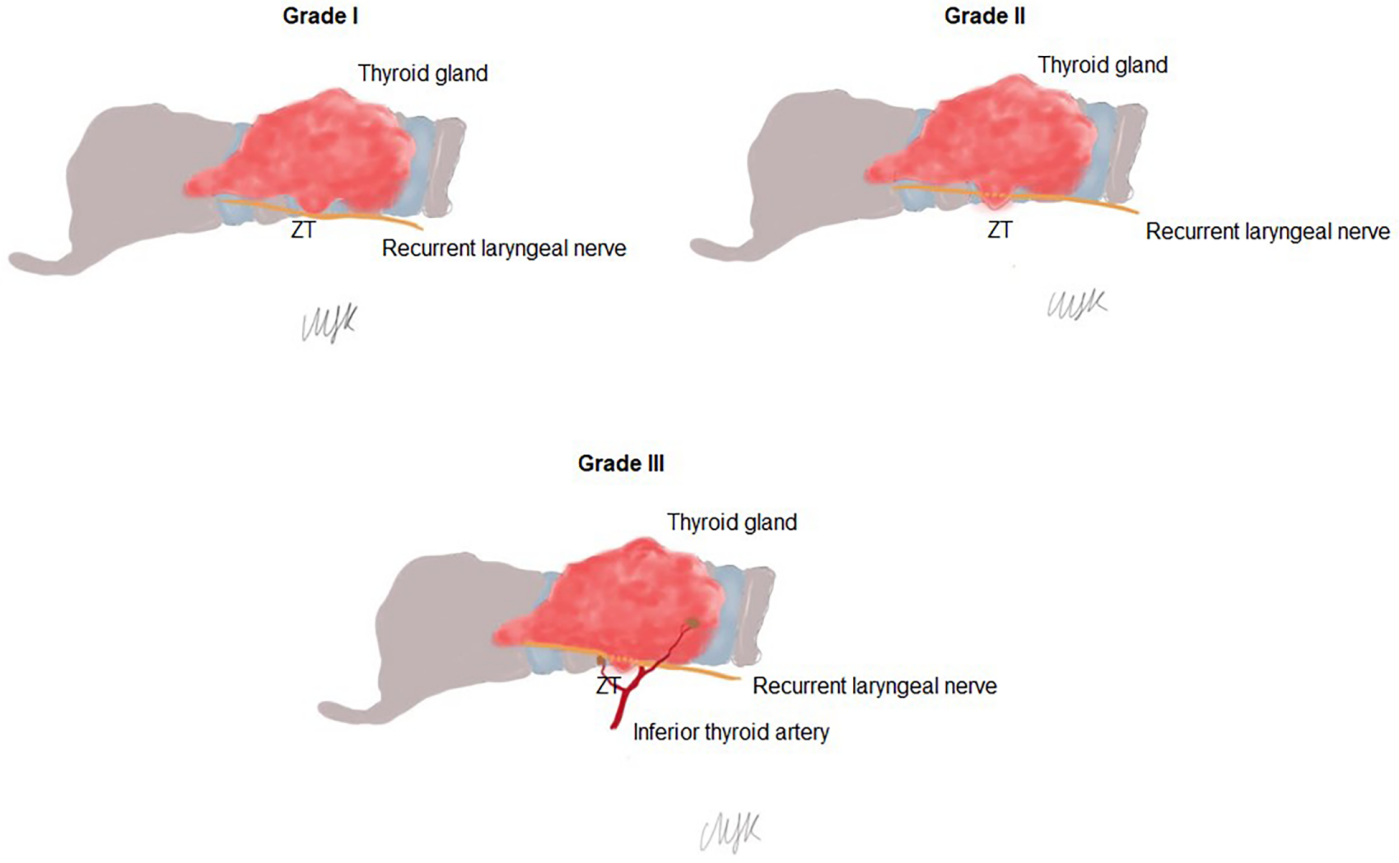
Grading of to Zuckerkandl's tubercle (ZT): Grade 1 (slight thickening of lateral edge of thyroid lobe), Grade 2 (less than 1 cm) and Grade 3 (more than 1 cm).

ZT grade was assessed using the grading system described by Pelizzo et al. (18), including Grade 0 (unrecognizable), Grade 1 (slight thickening of lateral edge of thyroid lobe), Grade 2 (less than 1 cm) and Grade 3 (more than 1 cm). The laterality of ZT was recorded in those classified as Grade I–III (recognizable) ZTs (Figure 1).

### 2.4. The relation between RLN and ZT

The running pathway of the RLN relative to ZT area was classified as medial to ZT, lateral to ZT and posterior to ZT.

### 2.5. The relation between SPG and ZT

**Figure 2:**
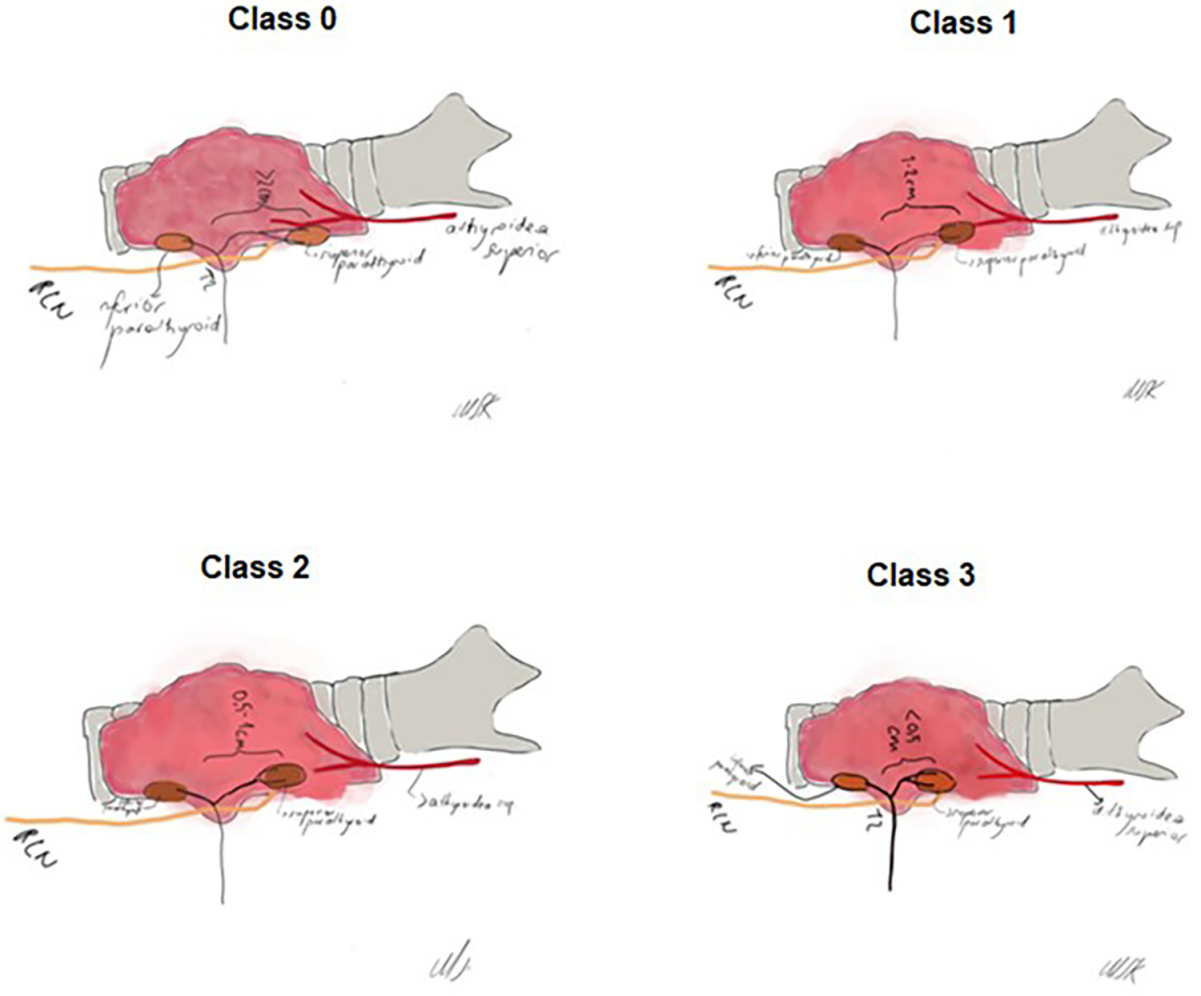
The course and position of superior parathyroid gland relative to Zuckerkandl's tubercle (ZT) as an anatomical landmark. Class 0 (2 cm from the ZT), Class 1 (1–2 cm from the ZT), Class 2 (0.5–1 cm from the ZT) and Class 3 (<0.5 cm from the ZT).

The relationship between SPG and ZT was assessed using the classification system described by Prakash et al. (17). The position of SPG relative to ZT was categorized as Class 0 (2 cm from the ZT), Class 1 (1–2 cm from the ZT), Class 2 (0.5–1 cm from the ZT) and Class 3 (<0.5 cm from the ZT) (17) (Figure 2).

### 2.6. Statistical analysis

Statistical analysis was performed using IBM SPSS Statistics (IBM Corp. Released 2012. IBM SPSS Statistics for Windows, version 22.0. Armonk, NY: IBM Corp). Descriptive statistics were reported including means and ranges for continuous variables and percentages for categorical variables. Data were expressed as mean (minimum–maximum) and percent (%) where appropriate.

## 3. Results

### 3.1. Patient demographics and thyroidectomy indications

Mean age of patients was 45.6 years (range, 18–78 years), 320 (76.2%) of 421 patients were females. Most of the thyroidectomy indications (69.1%) were related to malignant disease including papillary carcinoma in 54.9% of cases (Table 1).

**Table 1:** Patient demographics and thyroidectomy indications.

Age (year), mean (min–max)	45.6 (18–78)
Gender, *n* (%)	
Female	320 (76.2)
Male	101 (23.8)
Thyroidectomy indications	
Benign	130 (30.9)
Colloid goiter	27 (6.4)
Multinodular goiter	75 (17.8)
Adenoma	28 (6.7)
Malignant	291 (69.1)
Papillary carcinoma	231 (54.9)
Follicular neoplasia	35 (8.3)
Hurtle cell carcinoma	18 (4.3)
Medullary carcinoma	7(1.7)

### 3.2. Presence, grading and laterality of Zt

**Figure 3:**
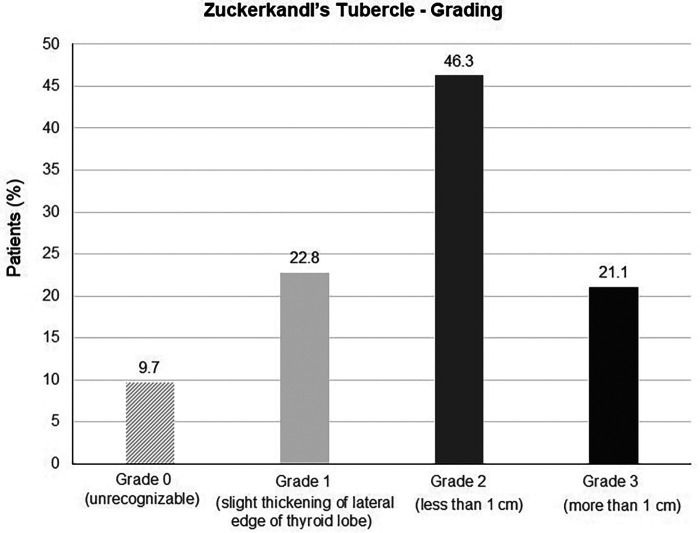
Zuckerkandl's tubercle grading in total thyroidectomy patients.

The ZT was unrecognizable in 41 (9.7%) of 421 patients. In 380 patients with identifiable ZT, grade 2 (46.3%) ZT was the most common finding, followed by the grade 1 (22.8%) and grade 3 (21.1%) ZTs (Table 2, Figure 3).

**Table 2:** Presence, grading and laterality of Zuckerkandl's tubercle.

Zuckerkandl's tubercle characteristics	
Grading (*n* = 421), *n* (%)	
Grade 0 (unrecognizable)	41 (9.7)
Grade 1 (slight thickening of lateral edge of thyroid lobe)	96 (22.8)
Grade 2 (less than 1 cm)	195 (46.3)
Grade 3 (more than 1 cm)	89 (21.1)
Laterality (*n* = 380), *n* (%)	
Unilateral	353 (92.9)
Left-sided	124 (35.1)
Right-sided	229 (64.9)
Bilateral	27 (7.1)

Majority of ZTs (92.9%) were unilaterally located (right-sided: 64.9%; left-sided: 35.1%), while only 7.1% of ZTs were found to be bilateral (Table 2).

### 3.3. The relation of RLN to ZT

In majority of the cases the RLN was found to lie medial to ZT (83.2%), followed by lateral (12.7%) and posterior (3.9%) positions.

### 3.4. The relation of SPG to ZT

**Figure 4:**
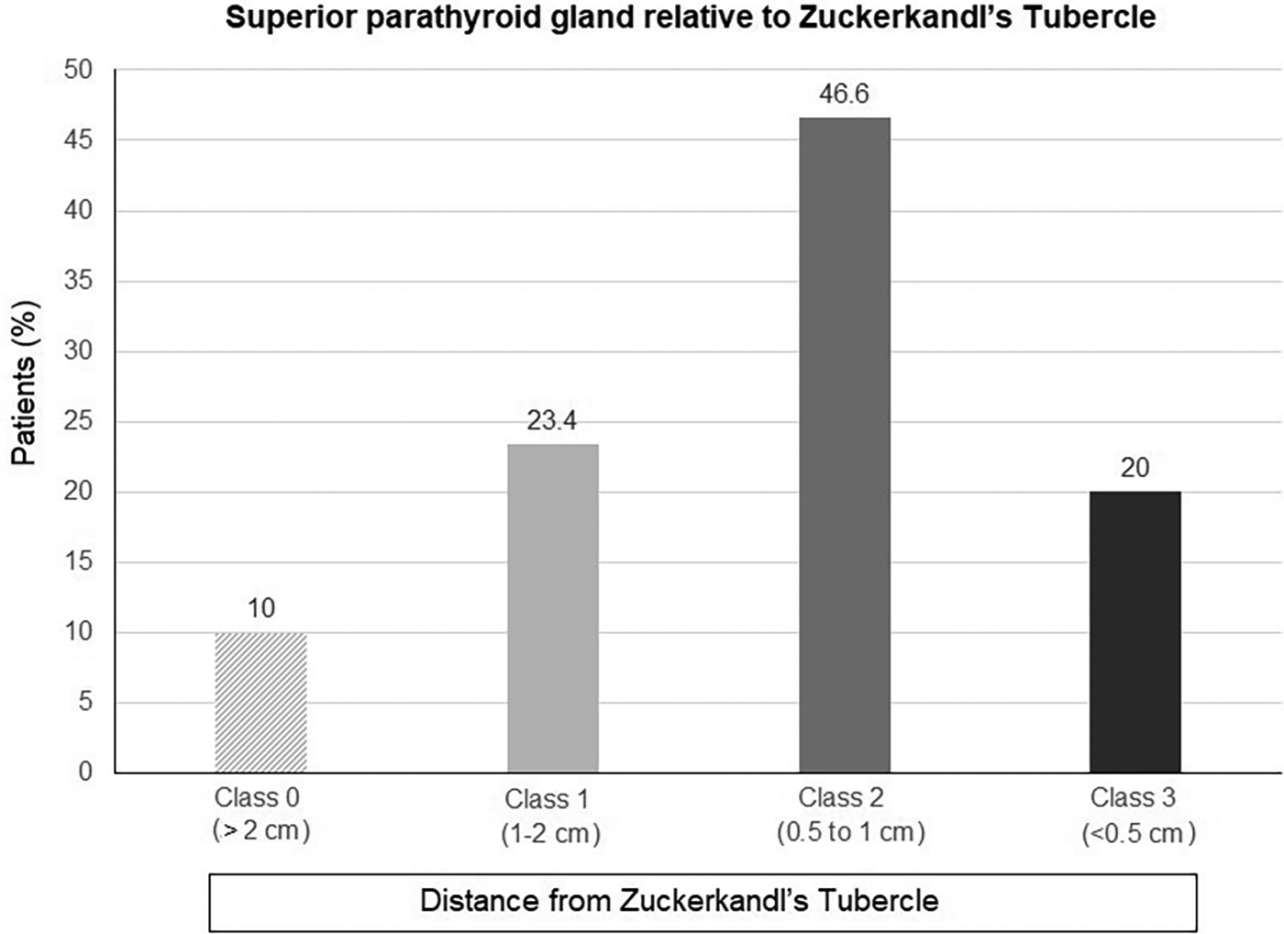
The relation between superior parathyroid gland and Zuckerkandl's tubercle in total thyroidectomy patients.

Overall, SPG was identified in close proximity to ZT in 66.6% of patients, including Class 2 (0.5–1 cm from ZT) pattern in 46.6% of patients and Class 3 (<0.5 cm from ZT) pattern in 20.0% of patients (Table 3, Figure 4).

**Table 3:** The relationship between SPG and ZT overall and by the ZT grading (*n* = 380).

	Total (*n* = 380)	Zuckerkandl's tubercle grade		
		Grade 1 (*n* = 96)	Grade 2 (*n* = 195)	Grade 3 (*n* = 89)
The distance of SPG from the ZT, *n* (%)				
Class 0 (>2 cm from the ZT)	38 (10.0)	20 (20.8)	11 (5.6)	7 (7.9)
Class 1 (1–2 cm from the ZT)	89 (23.4)	35 (36.5)	51 (26.2)	3 (3.4)
Class 2 (0.5 to 1 cm from the ZT)	177 (46.6)	28 (29.2)	111 (56.9)	38 (42.7)
Class 3 (<0.5 cm from the ZT)	76 (20.0)	13 (13.5)	22 (11.3)	41(46.1)

SPG, superior parathyroid gland; ZT, Zuckerkandl's tubercle.

**Figure 5:**
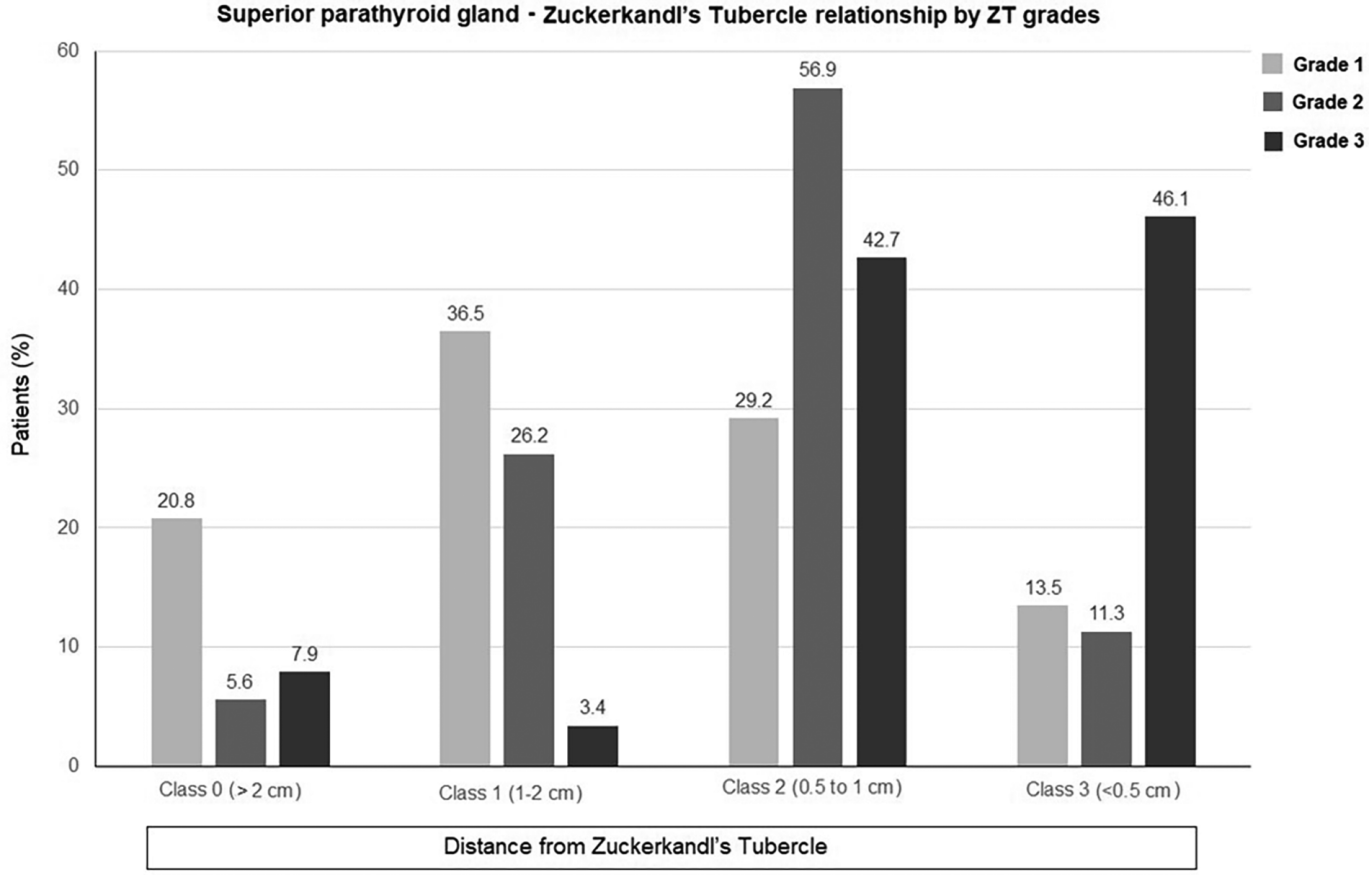
The relation between superior parathyroid gland and Zuckerkandl's tubercle by Zuckerkandl's tubercle grading in total thyroidectomy patients.

SPG was more likely to be identified in close proximity to ZT when the grade of ZT was higher. Accordingly, SPG was found to be located 0.5–1 cm from the ZT in 56.9% and 42.7% of grade 2 and grade 3 ZTs, respectively, and to be <0.5 cm from the ZT in 46.1% of grade 3 ZTs (Table 3, Figure 5).

## 4. Discussion

This prospective series of total thyroidectomies revealed that ZT was recognizable in majority of patients, as a landmark for locating both the SPG and RLN during the operation. ZT was predominantly unilateral with a predilection of a right-sided location in most of cases, and considered to be grade 2 in nearly half of patients. RLN was found to lie medial to ZT primarily. SPG was found to be in close proximity to ZT in most of patients, and the possibility of locating it nearby the ZT was increased by the advanced grade of ZT.

There is vast heterogeneity in the incidence of ZT reported in literature, which ranges from 7.0% to 90% in different thyroidectomy series depending on the geographical, genetic or ethnic factors as well as the use of accurate methodology in searching ZT during thyroidectomy (14, 17, 22, 23, 27–30). In our series, ZT was recognizable in majority (90%) of cases supporting its consideration of a constant landmark in locating RLN and SPG (14, 22, 23). Notably, in line with consistently reported predominance of grade 1–2 ZTs (18%–90%), nearly half of ZTs identified in our patients were grade 2 (<1 cm), which seems to emphasize the necessity for the surgeon's meticulous seeking and active effort to recognize ZT during the dissection (16, 17, 20, 21, 22, 23, 30–32).

In fact, the size of ZT is considered to be dependent not only on the amount of remnant lateral process tissue, but also on the pathology affecting the thyroid (17, 30, 33). The grade 3 ZT, with a reported incidence of 14% to 55% in thyroidectomy series, is suggested to be more commonly seen in enlarged thyroid glands with multinodular goitre, and considered to be a factor that can affect the completeness of thyroidectomy especially in less-experienced hands (14, 18, 20–24, 30). In this regard, the incidence of grade 3 ZTs (21.1%) in our patients seems consistent with the predominance of thyroidectomies performed for the malignant rather than benign indications in our series.

The predominantly unilateral location of ZT along with a two-fold higher incidence of right-sided vs. left-sided ZT (64.9% vs. 35.1%) in our patients support the data from previous thyroidectomy series indicated unilateral location of ZT in majority of patients along with a higher incidence on the right side (17, 21, 23, 26, 30–32). Likewise, in a series of 173 thyroid lobectomies from Turkey, ZT was reported to be recognizable in 73.4% of cases (grade 2 in 53.2%), and to be detected more frequently on the right vs. left side (78.2% vs. 68.6%) (17). In another study from Turkey in total and hemithyroidectomy series of 60 and 40 patients, respectively, ZT was identified in 66% of patients, and more commonly on the right vs. left side (61% vs. 38%) (21). The embryological difference of the two sides in relation to pharyngeal arches is considered to account for the more common presence of ZT on the right side, while the right-sided TZ is also suggested to be more consistently present in the setting of total thyroidectomy rather than the thyroid lobectomy (17, 32).

Theoretically, the ZT is considered to separate the parathyroid glands into the superior parathyroids and inferior parathyroids, and thus to serve as a pointer to SPG in thyroid surgery practice (18, 20, 30). In the current study, SPG was found to be in close proximity to ZT in most of patients, and the possibility of a closer relationship increased by the advanced grade of ZT. Similarly, in a study with 30 total thyroidectomy patients (10 with malignancies), SPG was found to be closely associated with ZT in majority of cases along with a strong relationship between the proximity of two structures and the ZT grade, indicating that the higher the grade of ZT, the closer the SPG to ZT (22). Also, in a study with 325 thyroidectomy patients (315 with malignancies), the authors noted that the smaller the size of ZT, the greater the distance between ZT and SPG (14).

Knowing that the SPG is more likely to be located in close proximity to a larger ZT seems to be a valuable information enabling the surgeon to take extra care when performing dissection around a larger grade ZT to preserve SPG (14, 22). Besides having a close proximity to the SPG, the larger ZT is also considered to make surgical dissection challenging at posterior site of the lateral lobes around the RLN and the inferior thyroid artery (21, 34).

The position of RLN relative to ZT in our patients support that RLN presents medial to ZT in majority of cases (80%–90%) and lies laterally in up to 10% (17, 20–22, 31, 35). Hence, ZT is considered a constant landmark pointing to the RLN, allowing easy identification of the nerve before it turns below the inferior cricothyroid articulation, if a careful and meticulous dissection is carried out around the ZT (17, 18, 23).

Majority of ZTs were unilaterally located in our series and was right-sided in most of cases. Nonetheless, ZT is considered a useful landmark to identify RLN bilaterally in studies reporting a higher incidence of bilateral ZTs (15%–25%, or even up to 90%) and/or equal incidence of right-sided and left-sided ZTs (14, 20–23). In addition, it should be noted that in thyroidectomies performed for malignant indications, RLN isolation may be challenging if the tumor extends to the ZT.

Albeit not detected in our series, NRLN is considered a rare but important anatomic variant associated with the five to six times higher risk of permanent nerve lesions in thyroid surgery (5, 6, 8). Indeed, the use of intraoperative neuro-monitoring (IONM) as an adjunct visual identification in thyroid surgery has been recommended, given that it allows the surgeon to recognize and differentiate true branches of RLN from sympathetic anastomoses, as well as the NRLN, during surgery (8, 36).

Overall, our findings indicate the utility of ZT as a reliable and constant anatomical landmark pointing to the RLN and the SPG during thyroidectomy, enabling to minimize the risk of inadvertent injury to the RLN as well the parathyroid injury (14, 17, 18, 21–27, 37). The careful and meticulous dissection around the tubercle thorough knowledge of the anatomy and embryogenesis of the thyroid gland is mandatory for safety of thyroid operations, given that the constant relationship of ZT with RLN and SPG may also cause a potential hazard for nerve and gland, or the presence a large ZT may also increase the risk insufficient surgery and complications, in the hands of inexperienced surgeons (1, 17, 18, 20, 21, 38, 39).

## 5. Conclusion

In conclusion, this prospective single-surgeon thyroidectomy series study indicates the likelihood of localizing the RLN medial to ZT, and the SPG in close proximity to ZT during total thyroidectomy operations. Hence, the ZT can be used as a reliable and constant landmark to localize both the RLN and the SPG, which enables minimizing the risk of iatrogenic injury to RLN while ensuring a parathyroid-sparing surgery with shorter operative time. Importantly, thyroid surgeons should have complete knowledge of thyroid gland anatomy and embryogenesis and should follow a careful and meticulous approach particularly for dissections around larger ZTs, given the increased likelihood of the SPG and the RLN to be in close proximity.

## Data Availability

The original contributions presented in the study are included in the article/Supplementary Material, further inquiries can be directed to the corresponding author.

## References

[1] Bhargav PR. Salient anatomical landmarks of thyroid and their practical significance in thyroid surgery: a pictorial review of thyroid surgical anatomy (revisited). Indian J Surg. (2014) 76:207–11. 10.1007/s12262-013-0856-x 25177118 PMC4141060

[2] Christou N Mathonnet M. Complications after total thyroidectomy. J Visc Surg. (2013) 150:249–56. 10.1016/j.jviscsurg.2013.04.003 23746996

[3] Patra A Asghar A Chaudhary P Ravi KS. Identification of valid anatomical landmarks to locate and protect recurrent laryngeal nerve during thyroid surgery: a cadaveric study. Surg Radiol Anat. (2023) 45:73–80. 10.1007/s00276-022-03054-y 36459179

[4] Stefanou CK Papathanakos G Stefanou SK Tepelenis K Kitsouli A Barbouti A Surgical tips and techniques to avoid complications of thyroid surgery. Innov Surg Sci. (2022) 7:115–23. 10.1515/iss-2021-0038 36561510 PMC9742273

[5] Toniato A Mazzarotto R Piotto A Bernante P Pagetta C Pelizzo MR. Identification of the nonrecurrent laryngeal nerve during thyroid surgery: 20-year experience. World J Surg. (2004) 28:659–61. 10.1007/s00268-004-7197-7 15175898

[6] Iorgulescu R Bistriceanu I Badanoiu D Calin C Capatana C Iordache N. Non-recurrent inferior laryngeal nerve: case report and review of the literature. J Med Life. (2014) 4(Spec Iss 4):90–4. PMC4813626 27057257

[7] Toniato A Merante Boschin I Pagetta C Casalide E Pelizzo M. A “pilot light” of the right non-recurrent laryngeal nerve, una spia del nervo non-ricorrente destro, surgical pathology, department of medical and surgical sciences, university of Padua, Italy. Acta Otorhinolaryngol Ital. (2010) 30:107–9. 20559482 PMC2882143

[8] Guerreiro S Lamas M Candeias H Eusébio R Rocha V. The non-recurrent laryngeal nerve: an anatomical “trap”. Rev Port Endocrinol Diabetes Metab. (2014) 9:84–7. 10.1016/j.rpedm.2014.05.001

[9] Thomas AM Fahim DK Gemechu JM. Anatomical variations of the recurrent laryngeal nerve and implications for injury prevention during surgical procedures of the neck. Diagnostics (Basel). (2020) 10:670. 10.3390/diagnostics10090670 32899604 PMC7555279

[10] Ngo Nyeki AR Njock LR Miloundja J Evehe Vokwely JE Bengono G. Recurrent laryngeal nerve landmarks during thyroidectomy. Eur Ann Otorhinolaryngol Head Neck Dis. (2015) 132:265–9. 10.1016/j.anorl.2015.08.002 26338514

[11] Mohebati A Shaha AR. Anatomy of thyroid and parathyroid glands and neurovascular relations. Clin Anat. (2012) 25:19–31. 10.1002/ca.21220 21800365

[12] Uludağ M Tanal M İşgör A. A review of methods for the preservation of laryngeal nerves during thyroidectomy. Sisli Etfal Hastan Tip Bul. (2018) 52:79–91. 10.14744/SEMB.2018.37928 32595378 PMC7315061

[13] Cho JN Park WS Min SY. Predictors and risk factors of hypoparathyroidism after total thyroidectomy. Int J Surg. (2016) 34:47–52. 10.1016/j.ijsu.2016.08.019 27554178

[14] Yun JS Lee YS Jung JJ Nam KH Chung WY Chang HS The Zuckerkandl’s tubercle: a useful anatomical landmark for detecting both the recurrent laryngeal nerve and the superior parathyroid during thyroid surgery. Endocr J. (2008) 55:925–30. 10.1507/endocrj.k08e-132 18566518

[15] Zuckerkandl E. Nebst bemerkungen uber die epithelkorperchen des menschen. Anat Hefte. (1902) LXI:61–82.

[16] Viveka S. Review of surgical anatomy of tubercle of Zuckerkandl and its importance in thyroid surgery. Chrismed J Health Res. (2018) 5:91–5. 10.4103/cjhr.cjhr_107_17

[17] Irkorucu O. Zuckerkandl tubercle in thyroid surgery: is it a reality or a myth? Ann Med Surg (Lond). (2016) 7:92–6. 10.1016/j.amsu.2016.03.030 27144005 PMC4840449

[18] Pelizzo MR Toniato A Gemo G. Zuckerkandl’s tuberculum: an arrow pointing to the recurrent laryngeal nerve (constant anatomical landmark). J Am Coll Surg. (1998) 187:333–6. 10.1016/s1072-7515(98)00160-4 9740193

[19] Chevallier JM Martelli H Wind P. Surgical discovery of parathyroid glands and the recurrent laryngeal nerve. Application of well known embryological concepts in the operating room. Ann Chir. (1995) 49:296–304. (French). 7668792

[20] Gauger G Delbridge LW Norm P. Incidence and importance of the tubercle of Zuckerkandl in thyroid surgery. Eur J Surg. (2001) 167:249–54. 10.1080/110241501300091363 11354315

[21] Gurleyik E Gurleyik G. Incidence and surgical importance of Zuckerkandl’s tubercle of the thyroid and its relations with recurrent laryngeal nerve. ISRN Surg. (2012) 2012:450589. 10.5402/2012/450589 22957274 PMC3431128

[22] Prakash BG Kamath KS Rajesh B Babu AR Sandhya D. Extended surgical implication of tubercle of Zuckerkandl in total thyroidectomy. Indian J Otolaryngol Head Neck Surg. (2021) 73:147–51. 10.1007/s12070-020-01854-5 34150588 PMC8163910

[23] Irawati N Vaish R Chaukar D Deshmukh A D'Cruz A. The tubercle of Zuckerkandl: an important landmark revisited. Indian J Surg Oncol. (2016) 7:312–5. 10.1007/s13193-015-0482-0 27651691 PMC5016326

[24] Yalcin B Poyrazoglu Y Ozan H. Relationship between Zuckerkandl’s tubercle and the inferior laryngeal nerve including the laryngeal branches. Surg Today. (2007) 37:109–13. 10.1007/s00595-006-3346-y 17243027

[25] Singh P Sharma K Agarwal S. Per operative study of relation of Zuckerkandl tubercle with recurrent laryngeal nerve in thyroid surgery. Indian J Otolaryngol Head Neck Surg. (2017) 69:351–6. 10.1007/s12070-017-1148-8 28929067 PMC5581774

[26] Gil-Carcedo E Menéndez ME Vallejo LA Herrero D Gil-Carcedo LM. The Zuckerkandl tubercle: problematic or helpful in thyroid surgery? Eur Arch Otorhinolaryngol. (2013) 270:2327–32. 10.1007/s00405-012-2334-7 23315185

[27] Page C Cuvelier P Biet A Boute P Laude M Strunski V. Thyroid tubercle of Zuckerkandl: anatomical and surgical experience from 79 thyroidectomies. J Laryngol Otol. (2009) 123:768–71. 10.1017/S0022215108004003 19000342

[28] Kaisha WA Saidi H. Topography of the recurrent laryngeal nerve in relation to the thyroid artery, Zuckerkandl tubercle, and berry ligament in Kenyans. Clin Anat. (2011) 24:853–7. 10.1002/ca.21192 21544871

[29] Gravante G Delogu D Rizzello A Filingeri V. The Zuckerkandl tubercle. Am J Surg. (2007) 193:484–5. 10.1016/j.amjsurg.2006.06.040 17368294

[30] Sheahan P Murphy MS. Thyroid tubercle of Zuckerkandl: importance in thyroid surgery. Laryngoscope. (2011) 121:2335–7. 10.1002/lary.22188 21898449

[31] Rajapaksha A Fernando R Ranasinghe N Iddagoda S. Morphology of the tubercle of Zuckerkandl and its importance in thyroid surgery. Ceylon Med J. (2015) 60:23–4. 10.4038/cmj.v60i1.7141 25804915

[32] Mehanna R Murphy MS Sheahan P. Thyroid tubercle of Zuckerkandl is more consistenly present and larger on the right: a prospective series. Eur Thyroid J. (2014) 3:38–42. 10.1159/000355823 24847464 PMC4005263

[33] Mirilas P. Grades of Zuckerkandl’s tubercle in normal thyroids. Surg Today. (2007) 37:918; (author reply 919–20). 10.1007/s00595-007-3510-z 17879048

[34] Freitas CAF Paroni AF Santos AN Silva RJSD Souza RO Silva TFD Can the Zuckerkandl tubercle assist in the location of the inferior laryngeal nerve during thyroidectomies? Rev Col Bras Cir. (2019) 46:e2249. 10.1590/0100-6991e-20192249 31508736

[35] Pradeep PV Jayashree B Harshita SS. A closer look at laryngeal nerves during thyroid surgery: a descriptive study of 584 nerves. Anat Res Int. (2012) 2012:490390. 10.1155/2012/490390 22737584 PMC3378964

[36] Donatini G Carnaille B Dionigi G. Increased detection of non-recurrent inferior laryngeal nerve (NRLN) during thyroid surgery using systematic intraoperative neuromonitoring (IONM). World J Surg. (2013) 37:91–3. 10.1007/s00268-012-1782-y 22955954

[37] Bagadiya P Damor K. A prospective study: Zuckerkandl tubercle an important anatomical landmark in identification of recurrent laryngeal nerve in thyroid surgery. Int J Surg Sci. (2020) 4:130–3. 10.33545/surgery.2020.v4.i3b.480

[38] Koçak S Aydintuğ S. Zuckerkandl’s tuberculum. J Am Coll Surg. (2000) 190:98–9. 10.1016/s1072-7515(99)00231-8 10625242

[39] Hisham AN Aina EN. Zuckerkandl’s tubercle of the thyroid gland in association with pressure symptoms: a coincidence or consequence? Aust N Z J Surg. (2000) 70:251–3. 10.1046/j.1440-1622.2000.01800.x 10779054

